# Finding the True Responders: Stratifying dMMR/MSI-H Tumors for ICI Response

**DOI:** 10.3390/cancers18010018

**Published:** 2025-12-19

**Authors:** Nari Kim, Seongwon Na, Jisung Jang, Mihyun Kim, Jun Hee Pyo, Kyung Won Kim

**Affiliations:** 1 Biomedical Research Center, Asan Institute for Life Sciences, Asan Medical Center, Seoul 05505, Republic of Korea; nari.kim.0908@gmail.com; 2 Departments of Radiology and Research Institute of Radiology, Asan Medical Center, College of Medicine, University of Ulsan, Olymphic-ro 43 Gil 88, Songpa-gu, Seoul 05505, Republic of Korea; securityin4@naver.com; 3 Trial Informatics Inc., Seoul 05544, Republic of Korea; etmira8787@gmail.com (J.J.); mihyun.kim@trialinformatics.com (M.K.); 4 Convergence AI Institute for Drug Discovery, Korea Pharmaceutical and Bio-Pharma Manufacturers Association, Seoul 06666, Republic of Korea

**Keywords:** dMMR, MSI-H, immune checkpoint inhibitors, immunogenomic stratification, biomarker, transcriptomic signature, precision immunotherapy

## Abstract

**Background/Objectives**: Immune checkpoint inhibitors (ICIs) show durable efficacy in tumors with deficient mismatch repair (dMMR) or high microsatellite instability (MSI-H), yet clinical responses remain heterogeneous. This study aimed to define immune subgroups within dMMR/MSI-H tumors and develop a reproducible transcriptomic signature predictive of ICI response. **Methods**: Four MSI-H-enriched cancer types (UCEC, COAD, READ, STAD) from The Cancer Genome Atlas were analyzed. Tumors were stratified by immune cell infiltration (MCP-counter immune composite score) and T-cell-inflamed gene expression profiles (GEP score). Integrating these two axes defined four immune subgroups. Differential expression, random forest feature selection, and pathway enrichment were performed to identify immune programs. A 20-gene immune signature representing the most immune-active subgroup was developed and validated across TCGA, GEO (GSE39582), and IMvigor210 cohorts. **Results**: Among the four subgroups, the most immune-active group showed strong activation of interferon signaling, antigen presentation, and T-cell-mediated pathways. The 20-gene signature—including CD74, STAT1, TAP1, and HLA-class genes—achieved high reproducibility (mean AUC = 0.95 ± 0.02; accuracy ≈ 89%). In the IMvigor210 cohort, this signature identified tumors with higher PD-L1 blockade response (55.6% vs. 32.8%, *p* = 0.034) and improved survival trends in the TMB-high subset. **Conclusions**: The proposed 20-gene signature quantitatively captures immune heterogeneity in dMMR/MSI-H tumors and serves as a practical, interpretable biomarker to identify true ICI responders and guide precision immunotherapy.

## Simple Summary

Immune checkpoint inhibitors (ICIs) have transformed cancer therapy, but not all patients with mismatch repair-deficient (dMMR) or microsatellite instability-high (MSI-H) tumors respond effectively. To explore this variability, we analyzed immune profiles of dMMR/MSI-H tumors and classified them into four immune subgroups. A distinct “HotHigh” group showed strong immune activation, from which we developed a 20-gene signature that accurately identifies patients most likely to benefit from ICIs. This signature was validated across multiple datasets, supporting its potential as a reliable biomarker to guide precision immunotherapy in clinical practice.

## 1. Introduction

Immune checkpoint inhibitors (ICIs), including antibodies targeting PD-1, PD-L1, and CTLA-4, have transformed cancer treatment by restoring cytotoxic T-cell-mediated antitumor immunity [1,2]. Despite their clinical success, only a subset of patients benefits from ICIs, underscoring the need for robust biomarkers to predict therapeutic response [3]. Tumors with high tumor mutational burden (TMB), abundant neoantigens, and increased immune infiltration generally exhibit higher immunogenicity and thus greater sensitivity to ICI therapy [4].

Deficient mismatch repair (dMMR) results from loss-of-function alterations in key MMR genes such as MLH1, MSH2, MSH6, and PMS2 [5], leading to the accumulation of insertion–deletion mutations at microsatellite regions—a condition known as microsatellite instability (MSI) [6]. Tumors with high MSI (MSI-H) or dMMR status exhibit a hypermutated phenotype with a large number of neoantigens, resulting in enhanced T-cell infiltration and a “hot tumor” immune microenvironment [7]. Consequently, MSI-H/dMMR tumors are highly responsive to immune checkpoint blockade, and based on this evidence, the U.S. FDA granted a tissue-agnostic approval of pembrolizumab for MSI-H/dMMR solid tumors in 2017, marking a milestone in precision oncology [8].

However, clinical outcomes among MSI-H/dMMR patients remain heterogeneous, with only a fraction achieving durable response to ICIs [9]. In the successful phase II KEYNOTE-158 trial, pembrolizumab demonstrated a robust objective response rate of approximately 30–35% in patients with MSI-H/dMMR non-colorectal cancers, including some achieving complete and durable responses [10]. Similarly, in the phase II CheckMate-142 trial, nivolumab in combination with low-dose ipilimumab achieved an objective response rate of 69% and a complete response rate of 13% in previously untreated MSI-H/dMMR metastatic colorectal cancer, with the median duration of response and survival not reached at a median follow-up of 29 months [11]. Despite these promising outcomes, a substantial subset of patients still exhibited limited or no response, indicating that MMR deficiency alone does not fully capture the immunogenic diversity underlying therapeutic sensitivity [12]. This underscores the need to identify additional immunogenomic determinants that define true responders among MSI-H/dMMR tumors [13]. Moreover, current clinical determination of MSI-H or dMMR status—primarily based on PCR for microsatellite instability or IHC for loss of MMR proteins—may be subject to false-positive or false-negative results, reflecting methodological and tumor heterogeneity [14,15]. Thus, beyond technical classification, a more refined and reproducible immunogenomic framework is needed to delineate truly immunogenic MSI-H/dMMR tumors and identify patients most likely to benefit from immune checkpoint blockade [16].

To address this unmet need, we aimed to develop a precise immunogenetic stratification framework to analyze intratumoral heterogeneity within dMMR/MSI-H tumors and identify patients most likely to benefit from ICIs. Specifically, we integrated immune cell infiltration (“hot” vs. “cold”) with T-cell-inflamed gene expression profiles (GEP-high vs. GEP-low) [17] to classify tumors into four immunological subgroups across four major MSI-H-enriched cancers. Differential expression genes (DEGs) and random forest-based feature selection were applied to identify key genes distinguishing these subgroups, followed by functional enrichment analysis to delineate the biological pathways underlying immune activation or suppression. Based on these analyses, the “Hot+GEP-high” phenotype—characterized by heightened immune signaling and effector T-cell activity—was defined as the most ICI-responsive subgroup. Using this framework, we derived a 20-gene immune signature characterizing the “Hot+GEP-high” phenotype and evaluated its ability to predict ICI response across independent datasets. The 20-gene signature was internally cross-validated in TCGA dMMR/MSI-H datasets and externally validated using the GEO (GSE39582) cohort, confirming its reproducibility and cross-platform consistency. Furthermore, to assess its clinical relevance, we performed an exploratory immunotherapy response evaluation using the IMvigor210 cohort, which demonstrated a significant association between the signature score and clinical outcomes, including objective response rate (ORR) and overall survival (OS).

This study overcomes the limitations of MMR status-based classification by introducing a data-driven framework that quantitatively captures immune heterogeneity within MSI-H/dMMR tumors. The proposed 20-gene HotHigh signature offers an evidence-based and reproducible approach to identify patients most likely to respond to immune checkpoint inhibitors, providing a practical tool to enhance precision immunotherapy and optimize patient selection in clinical settings.

## 2. Materials and Methods

### 2.1. Cohort Selection and Data Acquisition

We selected four tumor types for analysis: uterine corpus endometrial cancer (UCEC), colon adenocarcinoma (COAD), rectal adenocarcinoma (READ), and gastric adenocarcinoma (STAD). These four tumor types were prioritized because dMMR/MSI-H are relatively common in this setting, represent the tumor types for which PD-1 blockade has the strongest clinical evidence and regulatory approvals, and TCGA provides a large sample size with well-structured MSI/MMR annotations. This setting allows for identifying distinct immunogenomic features within dMMR/MSI-H tumors and distinguishing responder-associated profiles.

To define dMMR/MSI-H status within TCGA, we applied two complementary strategies. First, MSI annotations were obtained from TCGA clinical datasets via the UCSC Xena platform (UCSCXenaShiny R package) [18], and samples labeled as “MSI-High (MSI-H)” were included. Second, somatic mutation data were obtained from the TCGA Genomic Data Commons (GDC) Data Portal [19] and examined to identify tumors with truncating loss-of-function variants in MMR genes (MLH1, MSH2, MSH6, PMS2), including nonsense mutations, frameshift indels, splice-site alterations, or start codon disruptions. We combined samples that met both criteria to establish a dMMR/MSI-H cohort and downloaded their RNA-seq expression profiles from the TCGA Pan-Cancer Atlas. Specifically, we used the batch-corrected “EBPlusPlusAdjustPANCAN_IlluminaHiSeq_RNASeqV2” gene expression dataset, which has undergone empirical Bayes-based normalization to minimize cross-cancer batch effects. Data were retrieved via the UCSC Xena platform in July 2025.

### 2.2. Tumor Subgroup Classification

To characterize the immunological heterogeneity within the dMMR/MSI-H cohort, tumors were stratified based on two orthogonal axes: immune cell infiltration [20] and a T-cell-inflamed GEP [17].

The relative abundance of immune and stromal cell populations was quantified using the MCP-counter R package [20], which estimates cell-type-specific enrichment scores from bulk RNA-seq data based on 111 transcriptomic markers. Specifically, eight immune cell lineages (T cells, CD8^+^ T cells, cytotoxic lymphocytes, NK cells, B lineage, myeloid dendritic cells, monocytic lineage, neutrophils) and two stromal populations (fibroblasts, endothelial cells) were evaluated. Hierarchical consensus clustering (Euclidean distance, Ward linkage, k = 2) [21] was then applied to the MCP-counter matrix to categorize tumors with globally high versus low immune infiltration as “hot” and “cold”, respectively.

In parallel, the T-cell-inflamed GEP score was computed using a previously validated 18-gene signature associated with IFN-γ signaling, antigen presentation [17], and cytotoxic activity (e.g., CD8A, CXCL9, CXCL10, GZMB, IFNG, STAT1). For each sample, gene-level z-score normalization was performed, and the mean GEP score per sample was obtained. To stratify tumors into GEP-high and GEP-low groups, a two-component Gaussian mixture model (G = 2) was fitted as an a priori thresholding strategy, consistent with the bimodal gene expression framework [22].

By integrating these two dimensions—immune infiltration (hot/cold) and inflammatory gene expression (GEP-high/low)—tumors were categorized into four immunological subgroups: HotHigh, HotLow, ColdHigh, and ColdLow. By combining these two dimensions, tumors were categorized into four subgroups: Hot+GEP-high, Hot+GEP-low, Cold+GEP-high, and Cold+GEP-low, which are referred to as HotHigh, HotLow, ColdHigh, and ColdLow throughout this study.

### 2.3. Differential Expression and Feature Selection

To identify subgroup-specific gene sets, we applied a two-step strategy that combined DEG [23] and machine learning-based feature importance analysis using RF [24]. DEG analysis was performed by comparing each subgroup (Hothigh, HotLow, ColdHigh, and ColdLow) with the remaining samples. Genes were considered significant if they met the criteria of adjusted *p*-value < 0.05 (Benjamini–Hochberg correction) and |log2FC| > 1. In parallel, RF models were trained using the same subgroup classifications, and gene importance was assessed based on MeanDecreaseGini and MeanDecreaseAccuracy scores [25]. The intersection between DEG-identified and RF-important genes was defined as the preliminary candidate set, which served as the foundation for subsequent network-based prioritization [26]. This conservative approach ensured the selection of genes that reflect both statistically significant expression differences and predictive contribution, and these candidate genes were subsequently used for downstream analyses including PPI network construction, hub gene identification, and functional enrichment.

### 2.4. Functional Enrichment and Hub Gene Identification

#### 2.4.1. GO, KEGG, and Reactome Pathway Enrichment

Functional enrichment analysis focused on identifying immune-related pathways differentially enriched across the four subgroups. Processes related to antigen processing and presentation, T-cell activation, and lymphocyte differentiation were emphasized to enable functional comparison of tumor immune microenvironments between subgroups. Enrichment analyses were performed using the intersecting gene sets derived from DEG and RF results, implemented with the clusterProfiler R package [27] for Gene Ontology (GO) [28], Kyoto Encyclopedia of Genes and Genomes (KEGG) [29], and Reactome pathways [30]. GO enrichment was limited to the Biological Process (BP) category, as BP terms capture functional immune activities more directly than Molecular Function or Cellular Component categories. Significance was defined as an adjusted *p*-value < 0.05 using the Benjamini–Hochberg correction [31].

#### 2.4.2. Protein–Protein Interaction Network and Hub Gene Analysis

Protein–protein interaction (PPI) networks were constructed from the intersecting genes identified by DEG and RF analyses [32]. This step was performed to identify key regulatory genes within each subgroup. Networks were generated using the STRING database (Homo sapiens, confidence score > 0.7, experimental and curated evidence) [33] and visualized in Cytoscape [34,35].

Hub gene analysis was performed to select genes with the highest network centrality, representing core regulators within the PPI network [36]. Hub genes were prioritized using the CytoHubba plugin with the Maximal Clique Centrality (MCC) algorithm, and the top 10 genes from each subgroup were proposed as a representative biomarker panel [37]. Among these hub genes, those overlapping with top-ranked DEGs were retained for signature construction in the next step [37].

#### 2.4.3. Gene Set Enrichment Analysis (GSEA) of Immune Hallmark Pathways

GSEA was performed to characterize the immune microenvironment of each subgroup (HotHigh, HotLow, ColdHigh, ColdLow). Unlike the functional enrichment analyses that focused on the intersecting DEG–RF gene sets, GSEA requires genome-wide expression rankings; therefore, differential expression results for all genes were used to generate ranked gene lists, ordered by log2 fold change. Analyses were conducted with the fgsea R package using the MSigDB Hallmark gene sets, with a focus on immune-related pathways (e.g., interferon-γ response, interferon-α response, inflammatory response, allograft rejection) [38,39]. Statistical significance was defined as adjusted *p*-value (Benjamini–Hochberg) < 0.05.

Comparisons across the four subgroups focused on the relative enrichment of immune-activating pathways (e.g., IFN-γ response, T cell activation, inflammatory signaling) versus immune-suppressive or non-inflamed pathways (e.g., TGF-β signaling, EMT, angiogenesis) [40]. This analysis aimed to characterize distinct tumor immune environments associated with each subgroup. Leading-edge genes from these immune hallmark pathways were incorporated in the subsequent step to complement the hub gene set and capture immunologically active features specific to the HotHigh phenotype [41].

### 2.5. Identification of the ICI-Responsive Subgroup and Immune Signature Gene Set

To identify the subgroup potentially responsive to ICI, comparative functional and pathway analyses were performed across the four immune subgroups. Functional enrichment, hub gene mapping, and GSEA of immune hallmark pathways were integrated to assess overall immune activation and regulatory signaling patterns. The subgroup exhibiting the strongest immune-related transcriptional activity was regarded as potentially ICI-responsive for subsequent analyses.

The immune signature gene set was derived through a stepwise integrative approach combining differential expression analysis, pathway enrichment, and network analysis. First, differentially expressed genes (DEGs) between HotHigh and remaining subgroups were filtered using adjusted *p*-value < 0.05 and |log2 fold change| > 1. Second, gene set enrichment analysis (GSEA) was performed using the fgsea R package with MSigDB Hallmark gene sets, and leading edge genes were extracted from four immune-related pathways: interferon-gamma response, interferon-alpha response, inflammatory response, and allograft rejection. Third, hub genes identified from PPI network analysis (top 10 by MCC algorithm) were incorporated. The candidate gene pool was constructed by intersecting DEG-filtered genes with GSEA leading edge genes, combined with hub gene overlap. Final signature genes were selected by ranking candidates by |logFC| in descending order and selecting the top 20 genes. This ranking strategy was chosen to prioritize genes showing the most pronounced and consistent transcriptional upregulation in the HotHigh subgroup, while maintaining biological interpretability. The complete selection workflow with filtering criteria and gene counts is provided in Supplementary Table S1, and the R code for reproducibility is available in the code is available on GitHub (https://github.com/githubnsw/MSI-H-HotHigh-Classifier; accessed on 19 December 2025).

### 2.6. Predictive Model Development (20-Gene Signature Classifier)

We developed a logistic regression classifier to predict HotHigh tumors using the 20-gene signature in the discovery cohort (TCGA, n = 259). To account for differences in gene expression scales across samples, gene expression values were z-score normalized within the cohort prior to model training (mean = 0, standard deviation = 1 for each gene). The logistic regression model was trained using L2 regularization (C = 1.0) with a maximum of 1000 iterations [42]. Model coefficients were learned to maximize the likelihood of correct classification.

The final prediction model computes a linear combination of z-scored gene expression values weighted by learned coefficients [43]:

$$z=\beta^{0}+\sum_{i=1}^{20}\beta ᵢxᵢ$$

The probability of HotHigh classification is then calculated using the logistic (sigmoid) function:

$$P\left(HotHigh\right)=1/\left(1+e^{-z}\right)$$

Samples with P(HotHigh) ≥ 0.5 were classified as HotHigh.

### 2.7. Validation Framework

#### 2.7.1. Internal and External Validation

This cross-cohort validation framework was designed to test whether the classifier could (i) reproducibly identify HotHigh tumors within the discovery dataset and (ii) generalize effectively from RNA-seq to microarray data, demonstrating robustness to technical variations while preserving underlying biological signal integrity.

To implement this framework, two independent datasets were used. The TCGA discovery cohort (n = 259) served as the internal validation set to evaluate model reproducibility within the same RNA-seq platform using cross-validation. For external validation, the GEO dataset (GSE39582) was employed [44], comprising 585 colorectal cancer samples profiled on the Affymetrix HG-U133 Plus 2.0 microarray platform. Samples annotated as MSI-H were selected to construct an MSI-H-enriched external validation cohort comparable to the discovery dataset.

Both internal and external validations were performed using expression matrices filtered to the 20-gene signature used for model training. Each cohort was independently z-score normalized to prevent information leakage and to account for platform-specific expression distributions. The trained TCGA model (fixed coefficients and intercept) was directly applied to the normalized GEO dataset to compute HotHigh probabilities, with a predefined threshold of *p* ≥ 0.5 for classification.

#### 2.7.2. Immunotherapy Response Validation

To evaluate the clinical applicability of the 20-gene HotHigh signature, we performed an exploratory evaluation using data from the phase II IMvigor210 trial which enrolled patients with locally advanced or metastatic urothelial carcinoma treated with atezolizumab (anti-PD-L1) [45]. The dataset includes comprehensive clinical and molecular profiles, such as RNA sequencing data, ICI response, and TMB measurements, providing a well-characterized clinical setting to assess the predictive performance of the proposed 20-gene signature in association with real-world treatment outcomes.

We first examined whether the HotHigh signature correlated with ORR and OS across the entire IMvigor210 cohort (n = 234), followed by an analysis restricted to the TMB-high subset (≥10 mut/Mb, n = 97) to assess its predictive value among hypermutated tumors. Because MSI annotations were unavailable in IMvigor210, the TMB-high group was used as an exploratory surrogate for hypermutated or dMMR-like tumors, allowing us to investigate whether the signature could stratify responders within this biologically enriched subset. This two-level approach enabled evaluation of both the generalizability of the signature in an unselected cohort and its refined predictive value within a hypermutated population.

Gene expression data for the 20 signature genes were extracted from the IMvigor210 RNA-seq dataset and independently z-score normalized within the cohort (mean = 0, SD = 1 per gene). The fixed logistic regression model trained on the TCGA discovery cohort was directly applied to compute HotHigh probability scores, classifying samples as HotHigh (*p* ≥ 0.5) or Others (*p* < 0.5). Normalization was performed separately for each cohort to prevent information leakage and to account for cohort-specific technical variation, while model coefficients remained fixed to ensure reproducibility and generalizability across datasets.

ORR, defined as complete or partial response (CR/PR) versus stable or progressive disease (SD/PD) per RECIST v1.1, was analyzed using logistic regression. OS was evaluated using Kaplan–Meier estimation and Cox proportional hazards models. Differences in ORR between HotHigh and non-HotHigh groups were compared using Fisher’s exact test, and odds ratios (OR) with 95% confidence intervals (CI) were calculated. For OS, survival differences were assessed by the log-rank test, and hazard ratios (HR) with 95% CIs were reported. Statistical significance was defined as two-sided *p* < 0.05 (Benjamini–Hochberg correction), and all analyses were performed in R.

To further assess whether the HotHigh signature was independently associated with overall survival, multivariate Cox proportional hazards models were performed. Covariates included ECOG performance status, PD-L1 immune cell (IC) level, and metastatic disease status; tumor mutational burden (TMB) was additionally included in models fitted to the full cohort. Analyses were conducted using complete cases. These multivariate analyses were performed to distinguish prognostic effects from treatment-related predictive associations.

## 3. Results

### 3.1. Cohort Selection and Data Acquisition

A total of 259 dMMR/MSI-H tumor samples were accessed from publicly available TCGA datasets, representing 259 of 904 total cases (28.6%), and included in this analysis. These samples comprised UCEC (n = 131, 49.9%), STAD (n = 69, 23.6%), COAD (n = 50, 18.4%), and READ (n = 9, 11.8%) as summarized in Table 1. Gene expression values were obtained as TCGA-provided normalized expression data and subsequently log_2_-transformed [log_2_(x + 1)] prior to analysis.

### 3.2. Tumor Subgroup Classification

**Figure 1:**
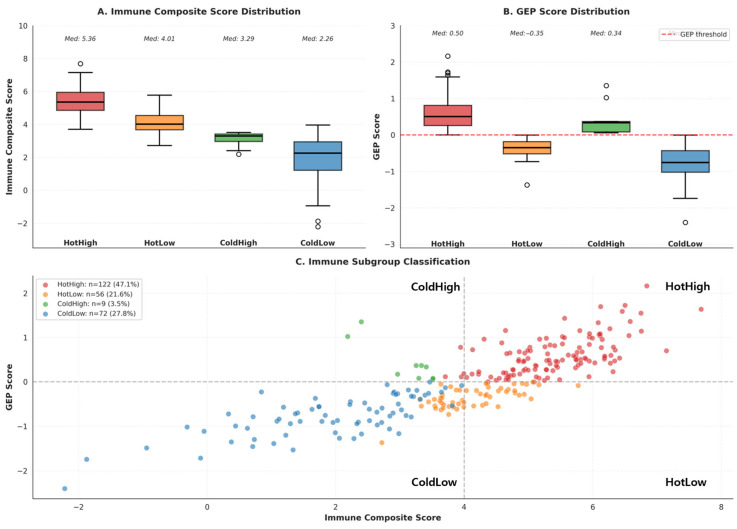
Immune Subgroup Classification in the TCGA dMMR/MSI-H discovery cohort. (**A**,**B**) Distribution of immune composite and GEP scores by subgroup. (**C**) Scatterplot showing four immune subgroups defined by immune composite and GEP scores.

Based on MCP-counter-derived immune cell abundance, 259 MSI-H/dMMR tumors were stratified according to global immune infiltration patterns. Hierarchical consensus clustering (Euclidean distance, Ward linkage, k = 2) identified 178 “hot” and 81 “cold” tumors, representing immune-inflamed and immune-desert phenotypes, respectively (Figure 1A). The overall silhouette score was 0.365, indicating moderate separation between clusters, with higher cohesion observed in the hot subgroup (average silhouette = 0.46) compared with the cold subgroup (0.14).

In parallel, a T-cell-inflamed GEP score was calculated for each tumor using a predefined 18-gene IFN-γ-associated signature. A Gaussian mixture model (G = 2) classified 131 tumors as GEP-high and 128 as GEP-low, corresponding to inflamed versus non-inflamed transcriptomic states (Figure 1B).

By integrating the two orthogonal dimensions—immune infiltration (hot/cold) and inflammatory gene expression (GEP-high/low)—four immunological subgroups were defined: HotHigh (n = 122, 47.1%), HotLow (n = 56, 21.6%), ColdHigh (n = 9, 3.5%), and ColdLow (n = 72, 27.8%) (Figure 1C). These subgroups were subsequently used as a framework for downstream analyses, including differential gene expression, pathway enrichment, and predictive model development.

### 3.3. Differentially Expressed Genes and Machine Learning Features

**Figure 2:**
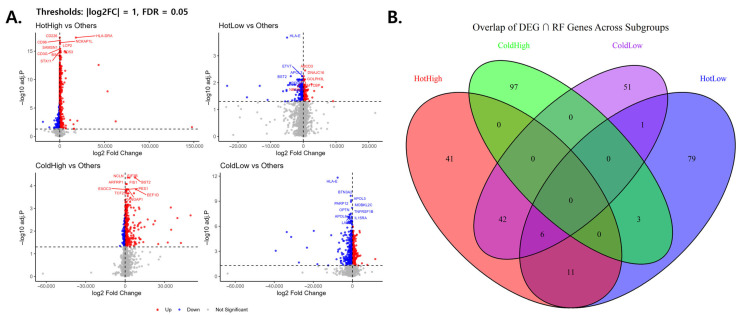
DEGs and their overlap with RF-selected features. (**A**) Volcano plots for each subgroup versus others. Red and blue dots indicate significantly up- and downregulated genes (|log2FC| > 1, adjusted *p* < 0.05), and the top 10 genes are labeled. In panel A, dashed vertical lines represent |log2 fold change| = 1 and dashed horizontal lines represent FDR = 0.05. (**B**) Four-way Venn diagram showing the overlap of genes jointly selected by differential expression and random forest analyses across immune subgroups. Limited overlap indicates subgroup-specific transcriptional features.

DEGs and RF feature selection were performed across the four subgroups. Volcano plots demonstrated distinct sets of up- and downregulated genes in each group. In the HotHigh group, immune activation-related genes such as HLA-DRA, LCP2, and CD226 were among the most significant, whereas HLA-E and BST2 were enriched in the HotLow group, EIF3B and BST2 in the ColdHigh group, and HLA-E, BTN3A2, and PARP12 in the ColdLow group (Figure 2A).

The overlap between DEG and RF-selected genes was further examined using a four-way Venn diagram (Figure 2B). It shows that genes jointly selected by differential expression and machine-learning importance are largely subgroup-specific, with no gene shared across all four immune subgroups, supporting distinct transcriptional programs underlying each phenotype. Subgroup-specific intersections were evident, with 41 genes in the HotHigh group, 97 in the ColdHigh group, 79 in the HotLow group, and 51 in the ColdLow group. Partial overlaps were also observed, including 42 and 11 genes shared between the HotHigh group and the ColdLow group, and 6 genes shared by the HotHigh group, ColdLow group, and HotLow group. Notably, no gene was common to all four subgroups.

### 3.4. Functional Enrichment

#### 3.4.1. GO, KEGG, and Reactome Pathway Enrichment

**Figure 3:**
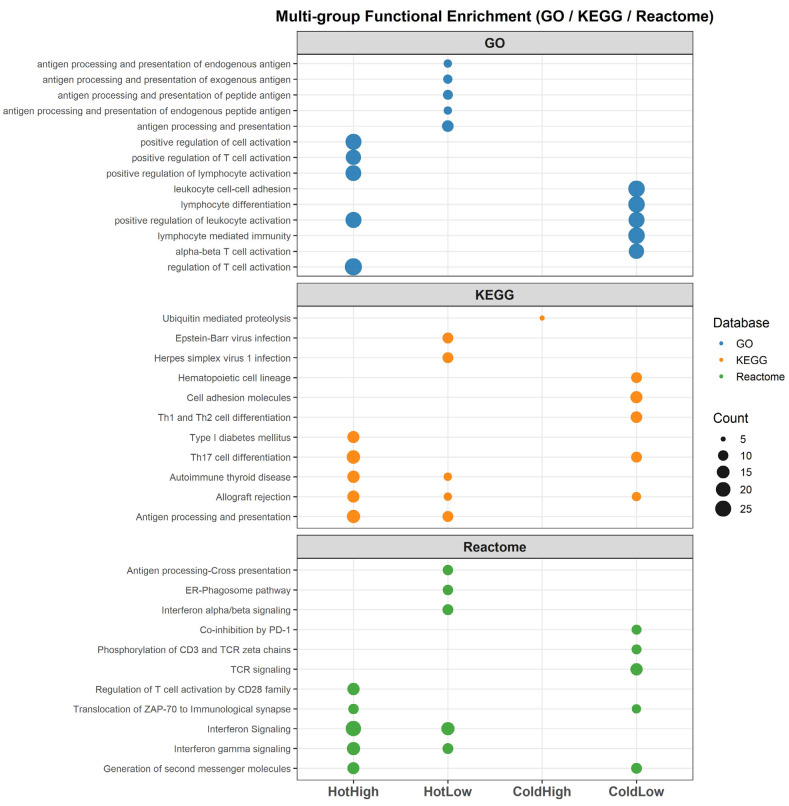
Multi-group functional enrichment analysis of intersecting DEGs and RF-selected genes. Dot plots show enriched pathways across four subgroups (HotHigh, HotLow, ColdHigh, ColdLow). Colors indicate the database source (blue = GO, orange = KEGG, green = Reactome), and dot size represents the number of genes involved in each pathway.

Functional enrichment analysis based on the intersecting gene sets from DEG and RF revealed distinct differences among the four subgroups (Figure 3). In the HotHigh group, pathways related to T-cell activation and inflammatory responses were prominent, including positive regulation of T cell activation, lymphocyte mediated immunity, Th1/Th2 differentiation, and TCR signaling. The HotLow group was characterized by repeated enrichment of antigen processing and presentation pathways, such as antigen processing and presentation and cross-presentation. In the ColdHigh group, immune-related pathways were largely absent, with only ubiquitin-mediated proteolysis detected. The ColdLow group showed enrichment of lymphocyte-associated programs related to differentiation and TCR signaling, together with PD-1-associated inhibitory pathways, but exhibits minimal enrichment of interferon-driven or cytotoxic immune responses. In contrast, E2F targets and cell-cycle pathways are prominent, defining ColdLow as a proliferative-cold tumor phenotype with attenuated immune effector activity.

#### 3.4.2. Protein–Protein Interaction Network and Hub Gene Analysis

**Figure 4:**
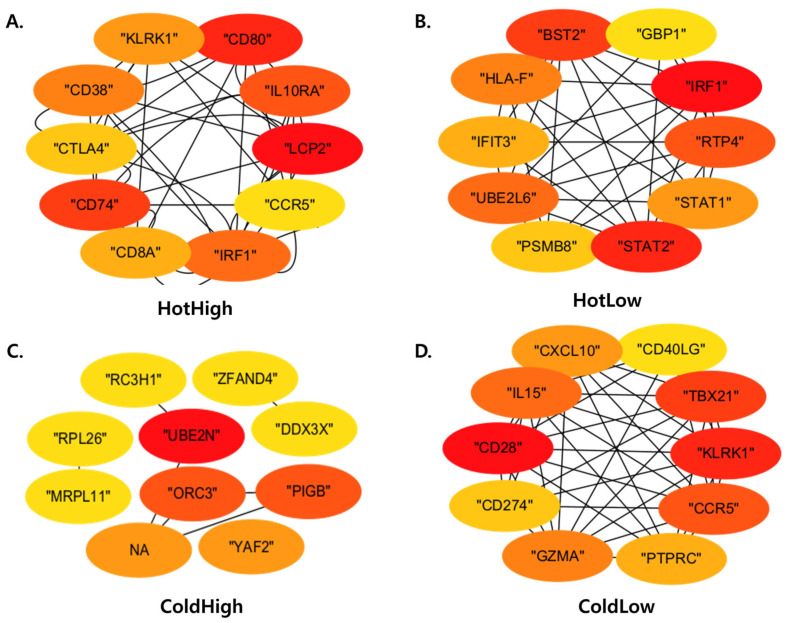
Protein–protein interaction (PPI) networks of the four immune subgroups. (**A**–**D**) PPI networks were generated for (**A**) HotHigh, (**B**) HotLow, (**C**) ColdHigh, and (**D**) ColdLow subgroups using the STRING database (confidence > 0.7). Node color indicates degree centrality (red = high, yellow = low).

PPI networks were constructed for the four subgroups, and network topology metrics were calculated (Supplementary Table S2). The HotHigh and ColdLow subgroups contained 88 and 87 nodes, respectively, with relatively high average degrees (8.14 and 12.60) and clustering coefficients, indicating dense interconnections among immune-related genes. By contrast, the ColdHigh subgroup showed the lowest connectivity, with 44 nodes, an average degree of 1.68, and a clustering coefficient of 0.256. The HotLow subgroup displayed an intermediate network size and density but remained less connected than HotHigh (Figure 4).

GO annotation of the top 25% hub genes ranked by MCC further highlighted subgroup-specific functional features (Table 2). In HotHigh, LCP2, CD80, and CD74 were enriched in positive regulation of protein phosphorylation, consistent with T-cell receptor signaling and co-stimulatory pathways. HotLow hub genes IRF1, STAT2, and BST2 were all associated with defense response to virus, reflecting an interferon-driven program. ColdHigh hub genes ORC3, PIGB, and UBE2N were linked to DNA replication initiation and ubiquitination, processes unrelated to immune activation. In ColdLow, CD28, KLRK1, and TBX21 mapped to positive regulation of lymphocyte-mediated immunity, indicating lymphocyte-associated regulatory and differentiation programs rather than effector immune activation.

#### 3.4.3. Gene Set Enrichment Analysis (GSEA) of Immune Hallmark Pathways

**Figure 5:**
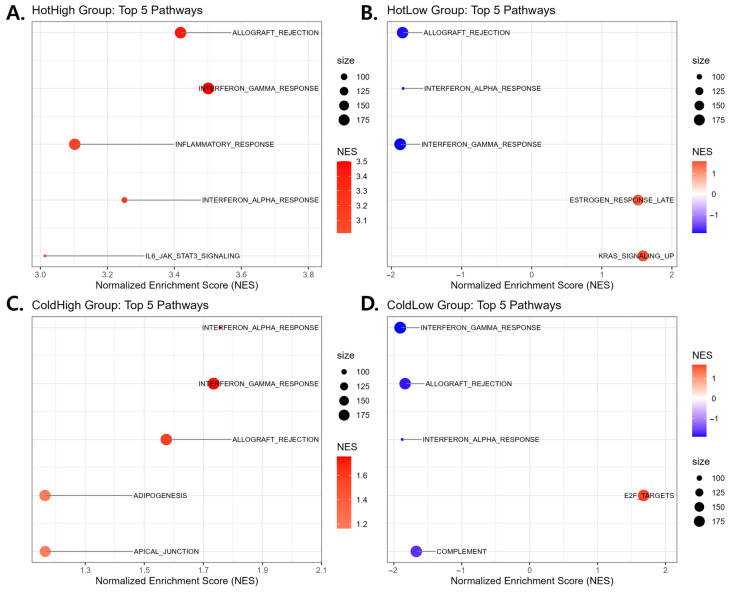
Gene Set Enrichment Analysis (GSEA) of the four immune subgroups. (**A**–**D**) Hallmark gene sets were analyzed with fgsea for (**A**) HotHigh, (**B**) HotLow, (**C**) ColdHigh, and (**D**) ColdLow subgroups. Each plot shows the top five significantly enriched pathways ranked by adjusted *p*-value. Dot color indicates the normalized enrichment score (NES; blue = negative, red = positive), and dot size represents gene-set size.

To explore the immune characteristics of the four subgroups, GSEA was performed using the Hallmark gene sets. Figure 5 highlights the top five pathways ranked by adjusted *p*-value, providing a clear comparison of representative features across groups.

The HotHigh subgroup exhibited marked enrichment of IFN-γ response, IFN-α response, inflammatory response, allograft rejection, and IL6–JAK–STAT3 signaling, confirming it as the most immune-active cluster. In contrast, the HotLow subgroup retained enrichment of IFN-γ/IFN-α response and allograft rejection, but with lower normalized enriched score (NES) values, and additionally showed estrogen response and KRAS signaling, reflecting partial immune activation together with cancer cell-intrinsic pathways. The ColdHigh subgroup also displayed enrichment of IFN-γ/IFN-α response and allograft rejection, although weaker than HotHigh, along with adipogenesis and apical junction, indicating metabolic and structural programs. The ColdLow subgroup showed limited enrichment of immune-related pathways, whereas E2F targets and cell cycle-associated programs were prominently enriched, suggesting an immune-cold, proliferation-dominant tumor phenotype. Full pathway enrichment results are provided in Supplementary Figure S1.

### 3.5. Identification of the ICI-Responsive Subgroup and Immune Signature Gene Set

Comparative multi-level analysis consistently identified the HotHigh group as the most immune-active phenotype (Table 3). This group was characterized by T-cell activation, lymphocyte-mediated immunity, Th1/Th2 differentiation, and TCR signaling, with hub genes such as LCP2, CD80, and CD74 indicating T-cell co-stimulatory functions. GSEA confirmed enrichment of immune-related hallmark pathways, including interferon-γ and interferon-α responses, inflammatory response, and allograft rejection. In the other groups, HotLow was defined by antigen processing and presentation, ColdHigh by DNA replication and ubiquitin-related processes, and ColdLow by proliferative programs with limited immune activity. These findings identify the HotHigh group as the principal subgroup associated with ICI response.

To derive a representative gene signature for the HotHigh phenotype, we applied a stepwise filtering approach integrating differential expression, pathway enrichment, and network connectivity (Supplementary Table S3). To derive a representative gene signature for the HotHigh phenotype, we applied a stepwise filtering approach integrating differential expression, pathway enrichment, and network connectivity (Supplementary Table S1). Differential expression analysis (HotHigh vs. rest) identified 2886 significant genes (adjusted *p*-value < 0.05, |log2FC| > 1). Leading edge genes from the four enriched immune Hallmark pathways yielded 227 core immune genes. Integration with 10 PPI hub genes produced a candidate pool of 182 genes. Ranking by |logFC| and selecting the top 20 genes yielded the final HotHigh immune signature: CD74, B2M, HLA-B, HLA-DRA, HLA-A, HLA-C, HLA-DRB1, HLA-E, STAT1, TAP1, CFB, IFITM1, RNF213, SOD2, IFI30, HLA-DQA1, SERPING1, C1S, IDO1, and CTSS. This signature is enriched for genes involved in MHC class I/II antigen presentation, interferon-gamma signaling, antigen processing, and complement cascade, collectively representing the transcriptional hallmarks of a T-cell inflamed tumor microenvironment.

### 3.6. Validation Framework

#### 3.6.1. Internal and External Validation

**Figure 6:**
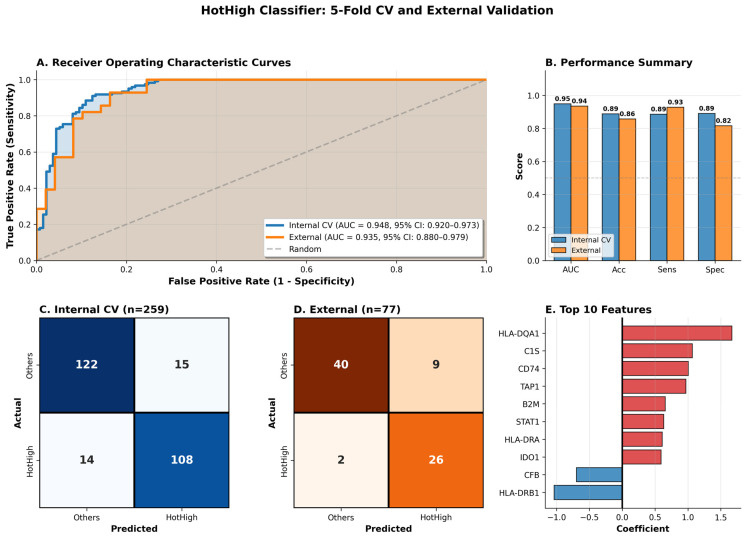
Cross-cohort validation of the 20-gene HotHigh classifier. (**A**) ROC curves for internal (TCGA) and external (GEO GSE39582) validations. (**B**) Summary of performance metrics (AUC, accuracy, sensitivity, specificity) across cohorts. (**C**,**D**) Confusion matrices for internal and external cohorts. (**E**) Top 10 model features ranked by logistic regression coefficient magnitude. Red bars indicate positive coefficients, and blue bars indicate negative coefficients.

To evaluate model reproducibility and prevent overfitting, 5-fold stratified cross-validation was performed on the TCGA cohort (n = 259). The classifier achieved a mean ± SD AUC of 0.948 ± 0.023 across folds (range 0.917–0.978; Figure 6A, Supplementary Table S1). Aggregated out-of-fold predictions yielded an overall AUC = 0.949, with accuracy = 88.8%, sensitivity = 88.5%, and specificity = 89.1% (Figure 6B). The corresponding confusion matrix (Figure 6C) shows that 108 of 122 HotHigh samples and 122 of 137 non-HotHigh samples were correctly classified. Performance consistency across folds (accuracy 82.4–94.2%; sensitivity 75.0–96.0%) demonstrates stable internal generalization.

For external validation, the model retrained on the entire TCGA dataset was evaluated in an independent cohort (GSE39582, n = 77) generated on the Affymetrix HG-U133 Plus 2.0 platform. Despite the platform difference, predictive power remained strong (AUC = 0.935), with accuracy = 85.7%, sensitivity = 92.9%, and specificity = 81.6% (Figure 6B). Among 28 HotHigh samples, 26 (92.9%) were correctly identified, and 40 of 49 non-HotHigh samples (81.6%) were correctly excluded, yielding PPV = 74.3% and NPV = 95.2% (Figure 6D). Examination of the model coefficients revealed that HLA-DQA1 showed the highest positive weight (1.67), followed by C1S, CD74, and TAP1, while HLA-DRB1 and CFB displayed negative coefficients (Figure 6E). The predominance of MHC class II and antigen-processing genes (HLA-DQA1, HLA-DRA, TAP1, B2M) highlights the importance of adaptive-immune and IFN-γ-related signaling in defining the HotHigh phenotype. Together, these results confirm that the 20-gene signature provides a reproducible, platform-agnostic, and biologically interpretable method for distinguishing HotHigh tumors, with robust performance across independent validation settings.

#### 3.6.2. Immunotherapy Response Validation

To determine whether the transcriptional HotHigh phenotype confers clinical benefit, we evaluated its association with response and survival in patients treated with PD-L1 blockade (IMvigor210 cohort). Analyses were performed in both the full cohort and the TMB-high subset to assess overall and hypermutated tumor contexts.

Across the full cohort, the ORR was higher in HotHigh tumors (27.3%, 30/110) than in Others (20.2%, 38/188), although the difference was not statistically significant (*p* = 0.198; OR = 1.48, 95% CI 0.85–2.57) (Figure S2A,B). The distribution of clinical responses (CR/PR vs. SD/PD) showed a similar trend, with 27.3% responders in the HotHigh group and 20.2% in Others. In terms of OS, median OS was 7.6 months for HotHigh tumors and 8.1 months for Others, with no significant difference between groups (log-rank *p* = 0.495; HR = 0.91, 95% CI 0.70–1.19) (Supplementary Figure S2C). These findings indicate that the HotHigh signature did not significantly distinguish response or survival outcomes within the unselected cohort.

**Figure 7:**
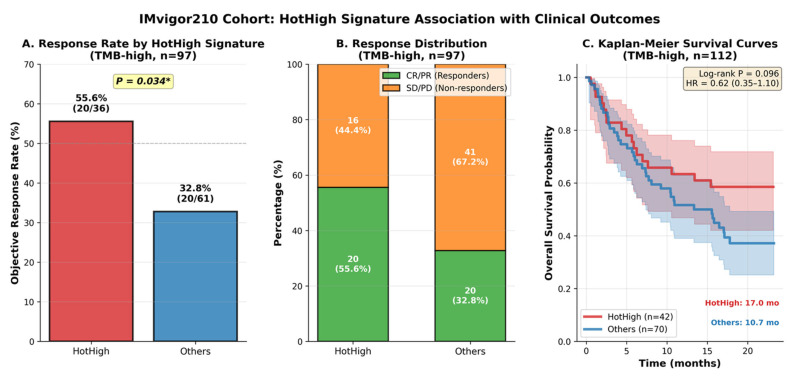
Association of HotHigh signature with immunotherapy response and survival in the IMvigor210 cohort (TMB-high subset). (**A**,**B**) HotHigh tumors exhibited significantly higher objective response rate (55.6% vs. 32.8%, *p* = 0.034; OR = 2.56, 95% CI: 1.10–5.98). (**C**) Kaplan–Meier overall survival showed a numerically favorable OS pattern in univariate analysis in the HotHigh group (median OS: 17.0 vs. 10.7 months; HR = 0.62, 95% CI: 0.35–1.10; log-rank *p* = 0.096). An asterisk (*) indicates statistical significance (*p* < 0.05).

Within the TMB-high subset, the HotHigh classifier effectively identified tumors with enhanced sensitivity to PD-L1 blockade. The ORR was significantly higher in HotHigh tumors (55.6%, 20/36) than in Others (32.8%, 20/61) (*p* = 0.034; OR = 2.56, 95% CI 1.10–5.98) (Figure 7A,B). HotHigh tumors thus exhibited nearly a two-fold enrichment of responders compared with non-HotHigh tumors. Consistent with the response data, HotHigh tumors in the TMB-high subset showed a numerically favorable overall survival pattern in univariate analysis, with a median OS of 17.0 months versus 10.7 months for Others (log-rank *p* = 0.096; HR = 0.62, 95% CI 0.35–1.10) (Figure 7C). However, this association was attenuated after adjustment for clinical covariates in multivariate Cox models, indicating that the HotHigh signature was not independently associated with overall survival.

While its predictive separation was limited in the unselected cohort, the HotHigh signature showed significant enrichment of responders in the hypermutated subgroup, underscoring its potential role as a predictive biomarker for immune checkpoint inhibitor response rather than a general prognostic marker.

## 4. Discussion

ICIs have been approved for use in tumors with dMMR or MSI-H, establishing one of the most robust biomarker-based treatment indications in oncology [12]. This approval, representing the first FDA tissue-agnostic indication based on a molecular biomarker rather than tumor site [8], marked a paradigm shift in precision oncology. Yet, in clinical practice, responses among dMMR/MSI-H patients are heterogeneous [46]. Some patients achieve long-lasting benefit from ICI therapy, whereas others show minimal or no response [9]. Clarifying the basis of this heterogeneity and identifying patients most likely to benefit remain critical unmet needs [47].

To address this heterogeneity, we stratified dMMR/MSI-H tumors into four distinct subgroups (HotHigh, HotLow, ColdHigh, and ColdLow) based on two complementary axes: immune cell infiltration [20] and the T-cell inflamed GEP [17]. This dual-axis framework enabled a systematic comparison of immune landscapes across subgroups. Among them, the HotHigh subgroup represented the most immune-active phenotype, characterized by strong activation of IFN and TCR signaling, co-stimulatory pathways, and antigen presentation machinery. From this subgroup, we derived a 20-gene HotHigh signature that consistently distinguished immune-inflamed tumors with high predictive performance. The final immune signature consisted of 20 genes, a scale consistent with previously validated transcriptomic biomarkers such as the 18-gene IFN-γ-related signature [48] and the Tumor Inflammation Signature [49], which balance interpretability and predictive stability in ICI response modeling. The model demonstrated strong reproducibility and generalizability, achieving a mean cross-validation AUC of 0.95 in the TCGA cohort and 0.94 in an independent GEO dataset, with accuracy and specificity exceeding 85%. These results confirm that the classifier effectively captures the transcriptomic features of immune-inflamed, IFN-γ-dominant tumors, independent of platform differences. In addition, we performed an exploratory assessment in the IMvigor210 cohort (atezolizumab-treated urothelial carcinoma) to examine whether the HotHigh gene expression signature is associated with clinical outcomes in an immunotherapy-treated setting. In this cohort, enrichment of responders and survival benefit was observed primarily within the TMB-high subset, whereas predictive separation was limited in the unselected population. Given the tumor-type mismatch and the absence of MSI annotation in IMvigor210, these findings should be interpreted as hypothesis-generating rather than as definitive external validation in MSI-H/dMMR cancers, and they do not support generalization to non-MSI-H tumors. Beyond identifying responder-enriched subgroups, our analysis also delineated populations with limited ICI sensitivity. The ColdLow subgroup was characterized by relative attenuation of immune-related signaling together with enrichment of cell cycle- and proliferation-associated pathways, including E2F targets, consistent with an immune-cold, proliferation-dominant tumor phenotype. Although lymphocyte-associated transcriptional programs were detectable, these features were not accompanied by enrichment of interferon-driven or cytotoxic immune pathways, suggesting that ICIs alone may be insufficient in this context. The predominance of tumor-intrinsic proliferative programs highlights potential opportunities for rational combination strategies, in which modulation of tumor growth-associated pathways or reconditioning of the tumor microenvironment may enhance responsiveness to immunotherapy.

This study has two main strengths: methodology and translational relevance. Methodologically, this study has three strengths. First, we applied a stepwise strategy that combined differential expression analysis with Random Forest-based feature importance, ensuring that selected genes were not only statistically significant but also informative for subgroup classification. Second, we reinforced the biological relevance of the candidate set through functional enrichment, network analysis, and hub gene prioritization, leading to a robust 20-gene panel. Third, we designed the analysis around four TCGA cohorts (UCEC, COAD, READ, STAD), where dMMR/MSI-H is relatively common and ICIs have the strongest clinical evidence, and we stratified tumors along two complementary axes—immune infiltration and a T cell-inflamed GEP—achieving a more refined classification than single-axis biomarkers such as TMB or GEP alone. Translationally, this framework refines patient stratification within dMMR/MSI-H tumors—providing a more precise, data-driven method to identify true responders to ICI therapy. The validated 20-gene HotHigh signature demonstrated strong reproducibility across independent datasets and sequencing platforms (AUC ≈ 0.95), suggesting that it can be feasibly implemented using existing transcriptomic assays (e.g., RNA-seq or targeted panels such as NanoString). Because the signature captures both adaptive and innate immune activation states, it could complement current biomarkers like PD-L1 IHC, MSI testing, or TMB, serving as an integrative transcriptomic biomarker to guide ICI selection. Moreover, the classifier’s binary output (HotHigh vs. non-HotHigh) can be easily interpreted in a clinical setting, facilitating patient stratification for immunotherapy trials or companion diagnostic development. In particular, its ability to distinguish immune-active and immune-silent phenotypes within dMMR/MSI-H tumors provides a framework for prioritizing patients likely to respond to ICIs or to benefit from rational ICI-based combinations.

The 20-gene HotHigh signature defined in this study clearly differs from previously reported T cell inflammatory-GEPs [17]. The T-cell inflamed GEP is primarily used as a molecular biomarker to identify T-cell-infiltrated tumors and to predict response to checkpoint inhibitors, being computed as a weighted sum of 18 genes related to IFN-γ signaling, cytotoxicity, and antigen presentation. While the GEP serves as a pan-cancer indicator of adaptive immune activation, our signature was specifically derived from dMMR/MSI-H tumors to capture the unique immunogenomic context of this population. Six genes (CD74, HLA-DQA1, HLA-DRB1, HLA-E, STAT1, IDO1) are shared between both panels, reflecting key pathways of the ICI response, including antigen presentation, interferon signaling, and immunoregulation. However, the T cell inflammatory GEP primarily emphasizes adaptive immune activity and includes genes such as CXCL9, CCL5, CD8A, NKG7, LAG3, and TIGIT, which represent T cell effector functions, chemokine signaling, and inhibitory receptors. In contrast, our signature describes a broader range of immune processes, including complement components (CFB, SERPING1, C1S), lysosomal protein degradation (IFI30, CTSS), and metabolic or oxidative stress-related pathways (SOD2, RNF213). In this way, the incorporation of adaptive and innate immune elements enables a broader characterization of tumor-immune dynamics within MSI-H/dMMR tumors, while also supporting a more consistent prediction of ICI responsiveness across heterogeneous tumor settings.

Several limitations should be noted. First, the silhouette score of 0.365 obtained from consensus clustering indicates modest cluster separation, reflecting the biological reality that immune phenotypes within dMMR/MSI-H tumors likely exist along a continuum rather than as strictly discrete states. Importantly, despite this moderate separation, the identified subgroups showed consistent and convergent differences across multiple analytical layers, including differential gene expression, pathway enrichment, protein–protein interaction network topology, and gene set enrichment analysis, supporting their biological relevance. Additionally, random forest-based feature importance estimated by MeanDecreaseGini may be influenced by gene expression variance. To address this limitation, RF importance was not used as a standalone selection criterion; instead, candidate genes were required to satisfy both differential expression significance and high RF ranking. The stability of the resulting gene set across independent cohorts and expression platforms further supports that the selected features capture biologically meaningful signals rather than variance-driven artifacts.

Second, this study was based on retrospective, publicly available datasets that differ in clinical context—TCGA primarily includes resected, treatment-naïve tumors, whereas IMvigor210 represents advanced ICI-treated cases—which may limit direct clinical translatability. External validation was conducted in a single-tumor GEO cohort of modest size and an advanced urothelial carcinoma cohort, underscoring the need for larger, clinically matched validation studies. In particular, IMvigor210 is biologically distinct from MSI-H-enriched gastrointestinal and endometrial cancers, and MSI status is not available in that cohort; therefore, our use of the TMB-high subset served only as an exploratory surrogate for hypermutated tumors. Accordingly, extension of these findings beyond MSI-H/dMMR settings (or to non-MSI-H cancers) remains speculative and warrants validation in clinically matched MSI-H/dMMR immunotherapy cohorts. Additionally, the analysis relied solely on bulk transcriptomic data without multi-omics or spatial validation, which may obscure cell-type-specific immune mechanisms. Although the integration of four MSI-H/dMMR-enriched tumor types enhanced generalizability, it may have masked tumor-specific immune diversity. Despite these constraints, the 20-gene HotHigh signature demonstrated consistent predictive performance across heterogeneous datasets and platforms, supporting its robustness and highlighting its potential for future clinical and experimental validation as a companion diagnostic biomarker.

Third, while the HotHigh signature showed significant predictive value for immune checkpoint inhibitor response in the TMB-high subset (ORR 55.6% vs. 32.8%; *p* = 0.034), the univariate survival advantage (HR 0.62) was attenuated after adjustment for clinical covariates (HR 0.87; *p* = 0.665). The wide confidence intervals likely reflect the limited sample size of the TMB-high subset (n = 112) and the exploratory nature of this analysis. Accordingly, these survival findings should be interpreted cautiously. The primary clinical utility of the HotHigh signature may therefore lie in predicting treatment response rather than long-term survival outcomes. 

Although the present study focused exclusively on dMMR/MSI-H tumors for analytical consistency, preliminary observations suggest that certain pMMR tumors may exhibit HotHigh-like transcriptional and immunologic features. Future studies integrating pMMR cohorts and combinatorial ICI regimens (e.g., lenvatinib plus pembrolizumab) may further expand the clinical utility of the HotHigh signature beyond canonical dMMR/MSI-H settings, enabling a broader and more refined stratification of ICI-responsive patients.

In summary, we propose a 20-gene signature derived from the HotHigh subgroup that enables precise identification of true responders among dMMR/MSI-H tumors. By quantitatively resolving immune heterogeneity within this population, the HotHigh signature offers a practical and reproducible biomarker to guide patient selection and optimize immunotherapy strategies in clinical practice.

## 5. Conclusions

We stratified dMMR/MSI-H tumors into four immune subgroups and identified a distinct HotHigh subgroup characterized by strong immune activation and T cell-mediated signaling. From this subgroup, we derived a robust 20-gene signature—CD74, B2M, HLA-B, HLA-DRA, HLA-A, HLA-C, HLA-DRB1, HLA-E, STAT1, TAP1, CFB, IFITM1, RNF213, SOD2, IFI30, HLA-DQA1, SERPING1, C1S, IDO1, and CTSS—that consistently distinguished HotHigh tumors across independent validation cohorts.

This signature provides a practical and reproducible biomarker to identify patients most likely to benefit from immune checkpoint inhibitors and to guide more precise immunotherapy strategies in dMMR/MSI-H tumors.

## Figures and Tables

**Table 1:** Summary of dMMR/MSI-H samples included in the analysis across TCGA cancer types. Number and proportion of dMMR/MSI-H and total tumor samples from UCEC, STAD, COAD, and READ cohorts. Percentages indicate the proportion of dMMR/MSI-H tumors within each cancer type.

Cancer Type	dMMR/MSI-H Samples (%)	All Tumor Samples
UCEC	131 (49.4%)	265
STAD	69 (23.6%)	292
COAD	50 (18.4%)	271
READ	9 (11.8%)	76
Total	259 (28.6%)	904

**Table 2:** GO enrichment of top 25% hub genes ranked by MCC across subgroups. Representative biological processes associated with subgroup-specific hub genes identified from the PPI network. *p*-values were adjusted using the Benjamini–Hochberg correction.

Subgroup	Gene	GO Annotation	*p*-Value
HotHigh	LCP2, CD80, CD74	positive regulation of protein phosphorylation	*p* < 0.01
HotLow	IRF1, STAT2, BST2	defense response to virus	*p* < 0.001
ColdHigh	ORC3, PIGB, UBE2N	DNA replication initiation	*p* < 0.05
ColdLow	CD28, KLRK1, TBX21	positive regulation of lymphocyte mediated immunity	*p* < 0.001

**Table 3:** Summary of subgroup-specific functional characteristics. Key biological processes, hub genes, and enriched Hallmark pathways defining each immune subgroup, with overall interpretation of immune phenotype.

Subgroup	Functional Enrichment	Hub Genes	GSEA (Hallmark)	Overall Interpretation
HotHigh	T cell activation, lymphocyte-mediated immunity, Th1/Th2 differentiation, TCR signaling	LCP2, CD80, CD74 (immune co-stimulatory)	IFN-γ, IFN-α, Inflammatory response, Allograft rejection	Immune-hot, ICI-responsive
HotLow	Antigen processing & presentation, cross-presentation	IRF1, STAT2, BST2 (interferon-driven)	IFN-driven + Estrogen/KRAS	Antigen-presentation-biased, partial immune activation
ColdHigh	Ubiquitin-mediated proteolysis only	ORC3, PIGB, UBE2N (DNA replication/ubiquitin)	IFN-driven + Adipogenesis	Minimal immune activity, metabolically driven
ColdLow	Lymphocyte differentiation, immune regulation	CD28, KLRK1, TBX21 (T/NK activation)	Weak immune; E2F targets, cell cycle	Immune-cold, proliferative

## Data Availability

All data used in this study are publicly available. TCGA datasets can be accessed via the Genomic Data Commons (https://portal.gdc.cancer.gov/ (accessed on 1 July 2025).), GSE39582 is available through the NCBI GEO database (https://www.ncbi.nlm.nih.gov/geo/), and the IMvigor210 cohort is available through the IMvigor210CoreBiologies R package (http://research-pub.gene.com/IMvigor210CoreBiologies (accessed on 1 July 2025).). The complete reproducible R code for gene signature derivation, model training and validation, together with the final gene list and model coefficients, is publicly available at the following GitHub repository: [https://github.com/githubnsw/MSI-H-HotHigh-Classifier (accessed on 1 July 2025)]. No new data were generated in this study.

## Supplementary Materials

The following supporting information can be downloaded at: https://www.mdpi.com/article/10.3390/cancers18010018/s1, Table S1: The 20-gene signature was derived through the following stepwise pipeline. Table S2: Topological properties of PPI networks across the four immune subgroups. Table S3: Performance Metrics of 5-Fold Cross-Validation for HotHigh Classifier on TCGA Discovery Cohort (n = 259). Figure S1: Full GSEA results of Hallmark pathways across the four immune subgroups. Figure S2. Association of HotHigh signature with response and survival in the full IMvigor210 cohort.
