# Supplementary material for: Finding the True Responders: Stratifying dMMR/MSI-H Tumors for ICI Response
Source: Cancers (Basel). 2025 Dec 19;18(1):18. doi: 10.3390/cancers18010018 (PMC12784724; doi:10.3390/cancers18010018)

Supplementary Materials

Table S1. The 20-gene signature was derived through the following stepwise pipeline:

Step	Description	Filtering Criteria	Genes (n)
1	DEG Analysis	HotHigh vs Rest, adj.P.Val < 0.05, |logFC| > 1	2,886
2	RF Feature Importance	Top 100 by MeanDecreaseGini	100
3	Hub Genes (PPI)	DEG ∩ RF → STRING PPI → MCC Top 10	10
4	GSEA Leading Edge	IFN-γ, IFN-α, Inflammatory, Allograft Rejection	227
5	Candidate Pool	(Step 1 ∩ Step 4) ∪ Step 3	182
6	Final Signature	Ranked by |logFC|, Top 20	20

The table outlines the sequential filtering steps used to derive the final 20-gene signature from the HotHigh subgroup. DEG: Differentially Expressed Gene; RF: Random Forest; PPI: Protein- Protein Interaction; MCC: Maximal Clique Centrality; GSEA: Gene Set Enrichment Analysis; logFC: log2 Fold Change.

Table S2. Topological properties of PPI networks across the four immune subgroups. Summary of network metrics including node count, edge count, density, average degree, and clustering coefficient for each subgroup.

Subgroup	Nodes	Edges	Density	Average degree	Clustering coefficient
**HotHigh**	88	358	0.094	8.14	0.501
**HotLow**	46	131	0.127	5.70	0.482
**ColdHigh**	44	37	0.039	1.68	0.256
**ColdLow**	87	548	0.146	12.60	0.545

Table S3. Performance Metrics of 5-Fold Cross-Validation for HotHigh Classifier on TCGA Discovery Cohort (n=259)

Fold	AUC	Accuracy	Sensitivity	Specificity	PPV	NPV
**Fold 1**	0.951	0.923	0.960	0.889	0.889	0.960
**Fold 2**	0.935	0.885	0.880	0.889	0.880	0.889
**Fold 3**	0.917	0.865	0.917	0.821	0.815	0.920
**Fold 4**	0.978	0.942	0.917	0.964	0.957	0.931
**Fold 5**	0.968	0.824	0.750	0.889	0.857	0.800
**Mean ± SD**	**0.950 ± 0.025**	**0.888 ± 0.047**	**0.885 ± 0.080**	**0.890 ± 0.051**	**0.879 ± 0.052**	**0.900 ± 0.061**

The table shows the performance metrics for each fold of the 5-fold stratified cross-validation. AUC: Area Under the Receiver Operating Characteristic Curve; PPV: Positive Predictive Value; NPV: Negative Predictive Value. Values are presented as proportions. Mean ± SD represents the average performance across all folds with standard deviation

Figure S1. Full GSEA results of Hallmark pathways across the four immune subgroups. Each dot represents a pathway, with color indicating normalized enrichment score (NES; red = positive, blue = negative) and size reflecting gene set size. Top five pathways per subgroup are summarized in Main Figure 5.

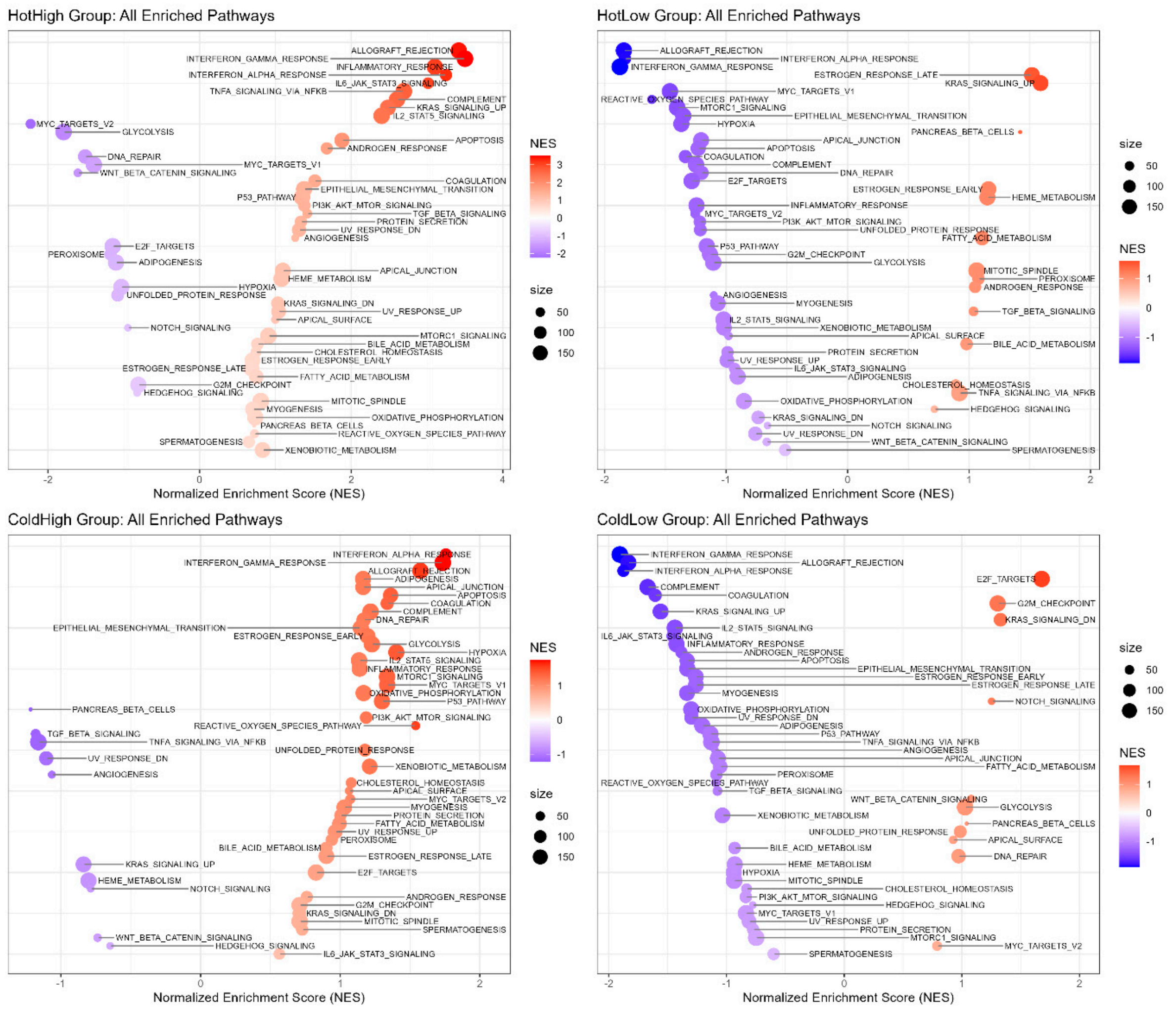

Figure S2. Association of HotHigh signature with response and survival in the full IMvigor210 cohort. (A–B) Objective response rate (ORR) was higher in HotHigh tumors compared with Others (27.3% vs. 20.2%, p = 0.198; OR = 1.48, 95% CI: 0.85–2.57). (C) Kaplan–Meier overall survival analysis showed comparable outcomes between groups (median OS: 7.6 vs. 8.1 months; HR = 0.91, 95% CI: 0.70–1.19; log-rank p = 0.495).

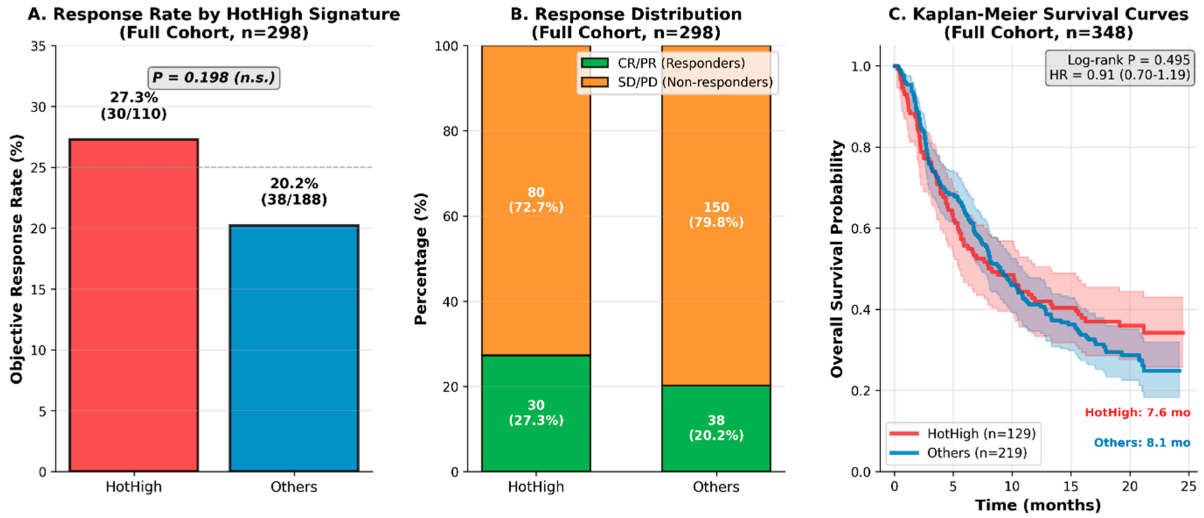

Supplement: Supplementary file 1 [file cancers-18-00018-s001.zip › cancers-4011718-supplementary.pdf]
